# Specification and production of prototype automatic instrumentation

**DOI:** 10.1155/S1463924679000237

**Published:** 1979

**Authors:** J. E. Carlyle

**Affiliations:** Department of Biochemistry Gartnavel General Hospital Glasgow G12 OYN Scotland


### Specification and production of prototype automatic instrumentation

**J. E. Carlyle**
*Department of Biochemistry, Gartnavel General Hospital, Glasgow, G12 0YN.*

### Introduction

In many laboratories staff salaries and overheads represent more than 70% of the total laboratory costs. The advent of automatic chemical analysis has permitted laboratories to accommodate large increases in workloads without proportional increases in staff numbers. In addition, levels of inaccuracy and imprecision are more easily standardised on automatic systems than on labour intensive manual methods.

The needs of chemical laboratories vary enormously. Certain tests are requested in large numbers and require a rapid 'turn-round'. These tests are generally carried out on multi-channel analysers of high capacity, e.g. 300 samples per hour, and each 'channel' of the instrument is dedicated to the analysis of a single sample constituent. Other tests, of lower volume and priority, are processed on single channel instruments which may be converted to carry out different tests during a working period.

### Prototype instrumentation

Manufacturing industries have successfully identified the needs of the clinical, university and other laboratories, and have generally provided the instruments required.

Many commercial instruments today however are developments of prototype units produced by users in individual laboratories. These have either been developed as a research project or in response to a laboratory's needs to accommodate routine requests.

The design and production of prototype automatic instruments is a complex project involving a range of expertise. It is generally only necessary when a suitable instrument is not commercially available or if available instrumentation cannot be readily modified to meet the user's requirements. These situations will arise when new separating or measuring techniques are developed, for example ion selective electrodes, or when a number of new features or developments of an existing system are not readily incorporated into an existing instrument.

This paper attempts to outline the production of a working prototype analyser in a user's laboratory, the variety of expertise required, and some of the pitfalls which may be encountered.

### General specifications

Before the project can proceed, the potential user must define the basic criteria for the production of the proposed instrument. These may be divided into technical and managerial requirements.

1. Technical:
   a. The analyte(s) to be measured and whether the instrument is to be multi or single 'channel'. Special approaches e.g. separation techniques required in the chemical methods should be specified at this stage.
   b. The maximum rate of sample throughput required. The life of a new instrument may be assessed as 7-10 years after which it will require major overhaul or be superseded by advances in technology. An attempt to assess the workload near the end of the projected life of the proposed instrument should be made.
   c. The type and volume of specimen to be analysed.
   d. The maximum acceptable levels of inaccuracy and imprecision for each analyte.
   e. The maximum acceptable level of mechanical and chemical unreliability.
   f. The desired level of operator intervention, i.e. what level of process control, data capture and data processing is envisaged?
2. Managerial:
   a. The maximum laboratory floor area to be allocated to the instrument.
   b. The maximum capital and revenue expenditure allowed for the project.
   c. The maximum time allowed for production of a working analyser.

### Feasibility study

The development will require expertise in analytical chemistry, mechanical and electronic engineering and, depending on the complexity of data capture and processing, in computer software. The team will also require the incorporation of a significant amount of technical expertise. User representation on the team, probably as the chemical expertise, is essential to the success of such a project.

The first function of the design team should be to carry out a feasibility study to assess whether the general specification can be achieved. The process of automatic chemical analysis may be subdivided into the following basic functions:-

1. Presentation of sample for analysis — usually aspiration of a fixed volume from a sample container.
2. Sample preparation — dilution; purification, e.g. protein removal e.g. dialysis or centrifugation; separation of constituents, e.g. ion exchange chromatography.
3. Reagent addition(s).
4. Mixing.
5. Incubation — analyser controls temperature and time.
6. Transfer function(s) — e.g. sample to reaction tube, reaction mixture to measuring device.
7. Measurement — potentiometry, photometry, etc.
8. Data acquisition — production of a permanent record of measurement.
9. Data processing — calculation of concentration, report format, etc.
10. Laundry — preparation of reaction area to receive the next sample.

Literature searches and discussions with colleagues who have a specialised knowledge of certain aspects of automation will identify the problems and pitfalls in each of the areas of the proposed project and to what extent the technique will fulfil the requirement of the specification. Multi-test analysers often impose compromises in methodology, e.g. it may be difficult to incorporate an ion exchange chromotography stage in an instrument where the majority of tests are single or two reagent additions with short colour development times. Generally multi-channel analysers will operate at a single incubation temperature for all channels although this restriction is avoided in some developments. Analytical rate, precision of results, and reliability are likely to be interrelated. The achievable precisions of sampling and dispensing systems are published with instrument evaluation reports [1] and acceptable values for some measuring systems are also available [2]. Variance of results, induced by fluctuations in temperature and incubation times, are not always proportional to the magnitude of the variation. Assessment of such errors is more difficult and may require experimental work. Discrete chemical analysers can operate at high analytical rates with better carryover and precision characteristics than a continuous flow system. However, the small number of moving parts in the latter results in a system with good mechanical reliability, relatively minor production complexities and flexibility, for example a variety of detector systems may be added with minimal effort.

The cost and time limits imposed may not allow production of all sub units by the development team. The project may still be feasible however by incorporating suitable modules which are commercially available.

It will be difficult to project the final cost at this stage but certain areas of hardware, e.g. computing equipment, dispensing systems, etc. can be assessed from commercial suppliers of related modules and an approximate figure will suffice. Similarly, expertise should exist within the team to assess whether production of a working prototype within the time scale is reasonable.

### Detailed specifications

Production of a detailed specification is a vitally important stage of development. Features omitted at this stage may be difficult to incorporate later. The mechanical and chemical principles of operation should now be defined.

Details required at this stage are summarised below.

1. Methods:
   Sample and reagent volumes, number of reagent additions, incubation temperature and time, any special procedures for each method must be specified.
   The rate of reaction should be known such that the effects of temperature and timing variations can be assessed.
   Minimum and maximum absorbance or other signal should be stated for each method and these ranges should coincide with good signal to noise ratio levels for the detectors, e.g. absorbance values below 0.1A or above 2.0A should be avoided.
2. Sample/diluting system:
   The requisite accuracy and precision of these units must be stated for the range of volumes encountered in the range of methods.
   A comprehensive range of diluter and dispenser systems is commercially available; some with remote control of volume settings. It is unlikely that the development team would produce a system significantly different in performance and the incorporation of commercially available units is likely to be less expensive.
   The precision of dispenser systems improves with increasing volume settings, therefcre a compromise between sample volume and precision may be desirable.
3. Mixing/temperature control:
   Agitation of the reaction container, mechanical stirring or 'jet-mixing' will all provide adequate mixing under the correct conditions. The latter approach has the advantage of minimising mechanical complexity although addition of small volumes of 'starter reagent' to a large reaction volume will usually not provide adequate mixing. Agitation of the container or stirring may enhance temperature equilibration of the solution. Any temperature variation should be $\pm$ 0.1°C or less.
   Control systems may use air flow, oil or water baths, or solid state heating. The selection of the appropriate method will depend on the geometry of the reaction area although air heating will probably require fan(s) to maintain air flow. Proportional controllers should be used with all systems.
4. Detectors:
   Measurements may include photometric, potentiometric, or other devices. The measuring wavelength should be stated for each method of photometry employed and the allowable 'band-pass' should also be stated.
   The response of silicon photodetectors is satisfactory in the visible area of the spectrum while vacuum phototubes or photomultipliers will be required for measurement in the ultraviolet region.
   The complexity of a multichannel photometer may be reduced by providing a single light source and incorporating fibre optic light guides in the design. Acceptable 'noise' and 'drift' levels should be specified and these will be higher for photometric measurements incorporating a flame unit. The required characteristics of the detector response should be stated, i.e. the range of linearity and signal to noise ratio etc.
5. Data acquisition:
   Detector outputs will usually be in analogue form. The final output may be in analogue, e.g. chart recorder, or digital form. In the latter case in analogue to digital converter (ADC) will be required. The frequency of signal sampling will define the type of ADC required. A multiplexor may be used to sample all channels of a multi-test analyser sequentially and transmit the signals to one ADC. The digital value may be printed or entered to a computing unit for further processing. In the former case the rate of printing required should be stated and any requirements for data formatting should also be included.
6. Computing facilities:
   A detailed 'flow diagram' of all data input, storage, processing, and output is essential to specify hardware requirements. The flow-diagram should also detail other signal inputs or outputs which may be used for 'process control'.
   Hardware specifications will consider: core size, storage medium and capacity, programming language, input and output devices, power fail/restart, facility, real time clock. Many of the specifications will be interdependent, e.g. the use of a high level language such as FORTRAN will require extra core to store the compiler and additional input facilities may be required to load the compiler after computer malfunction. However if complex programs are envisaged additional capital costs of hardware and software for high level language facility may be offset by savings in staff time since the development stage timescale should be reduced.
7. Process control:
   An integral minicomputer or separate electronic control may significantly reduce the level of operator intervention. Facilities such as automatic 'shut-down' or 'stand-by', warning systems for low reagent levels or sub-unit failure should be considered.
8. 'Back-up' facilities:
   Modularity in instrument design allows easy replacement of sub-units when failure occurs. If a very low level of 'downtime' is expected of an instrument replacement of sub-units is essential.
   Certain sub-units, critical to machine operation, e.g. the computer, may not be easily replaced and it may be possible to design a 'back-up' output such as recorder, separate hard copy or punched paper tape. The machine may then continue to operate, at an acceptable level, until the fault is repaired.

### Design and production

The detailed specification delineates the technical details of the instrument. Commercially available sub-units should be selected and tested and those areas where special units have to be produced must be quickly identified.

### Ergonomics

The operation, maintenance, and repair of the instrument should be under constant assessment during the design stage. The incorporation of available sub-units may restrict the ergonomics of the design. The control panels and reaction area should be in close proximity and units removed or cleaned during routine maintenance should be readily accessible. Electrical and electronic sub-units should be rack mounted with 'plug-in' or edge connectors. Rack mounting of the computer is likely to be necessary for routine maintenance by the supplier.

### Safety

Biological, chemical, electrical, mechanical and noise hazards must all be considered during the design stage. Clinical laboratories in particular analyse biological materials which are potentially infective. Instrument design should aim at minimal aerosol formation or splashing in the sample processing area. Waste receptacles should have overflow protection devices to avoid spillage. Corrosive and potentially carcinogenic reagents and those producing inflammable vapours should be avoided.

Preliminary electrical safety requirements for laboratory equipment are currently under consideration [3] but considerations on electrical safety should include the following: Electrical

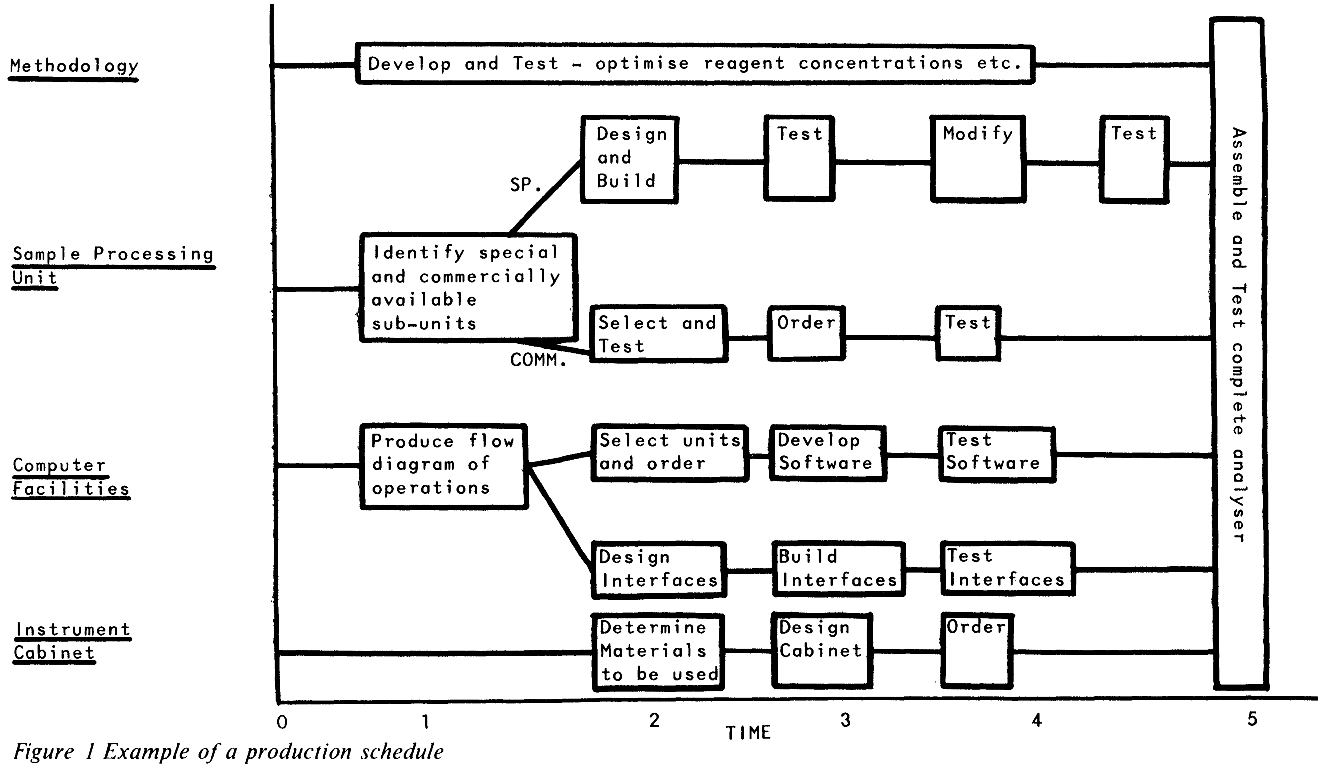

*Figure 1 Example of a production schedule*

mains cables should be appropriately colour coded according to local and international standard specification. All electrical harnessing should be colour coded and high voltage terminals should be isolated from hydraulic areas where possible. Electrical control circuits near liquid handling areas should be ot low voltage and electrical sparks for example from relays minimised by encapsulating the contacts. Earth continuity of all metal areas of the instrument should be tested after assembly.

Mechanical hazards caused by sharp metal edges must be avoided and any moving assemblies with high velocity ratios should be guarded. In addition, assemblies with extremes of temperature or pressure should be shielded from the operator.

High noise levels from cooling fans, printers or other components produce stress on the operator and instrument design should aim at minimum noise levels.

### Consumables

The instrument should where possible use existing consumables such as sample cups, reaction tubes, glassware etc. Moulding of special consumable items in low volumes will be expensive and should be avoided.

### Instrument cabinet

A variety of cabinet manufacturers will build a cabinet to the design team's specification. The following details will have to be provided: frame and panel materials including paint finish, dimensions, maximum loading on load bearing areas, positions of cooling louvres, specifications for doors or hinged panels.

### Production

A critical path analysis should be made of a detailed production schedule. From the example in Figure 1 it is clear that specially produced units will take longer to obtain or produce than those which are commercially available. Computer software development may continue after the detailed specification of the computer is known but program testing will be difficult before the computer is delivered unless the programmers share access to a similar computer. Other sub-units should be tested independently before assembly in the analyser if possible. Final assembly of the analyser will depend on the delivery of the instrument cabinet.

Quotations and delivery terms must be in writing for all components. Delivery will often be quoted as between 'x' to 'y' weeks and it is advisable to incorporate the 'y' figure in a critical path analysis.

### Testing and evaluation

After final assembly the design team should determine that the basic criteria of the project have been achieved. The prototype analyser may then be transferred to a user's laboratory for evaluation. The design team should prepare an operating and repair manual in accordance with agreed criteria [4] which should accompany the instrument.

The instrument should be evaluated over a period sufficient to allow a true assessment of the safety, reliability and performance levels. Agreed protocols, such as those outlined by Roberts [5], should be used in the evaluation.

### Discussion

Prototype is defined as 'the original from which others are copied' or 'an ancestral form'. The capital investment in a production prototype may be considerable. The salaries of the design team alone may exceed £20,000 per annum. Therefore classification of an instrument as a prototype should not be an excuse for production of a sub-optimal unit. It is inevitable that improvements to a prototype will be made, but these should occur as a result of operational factors which could not be easily predicted.

Many potential problems exist in production of prototype instrumentation but it should be emphasised that few specifications can be too detailed; it is simpler to remove extra specifications at a late stage in the development than to incorporate them. Commercially available components or sub-units should always be tested, preferably before purchase, to ensure that the requisite specifications are met. Finally the assembly of a prototype analyser, in a reasonable time, depends on a detailed schedule of functions each of which will take a finite time. A good critical path analysis may save weeks of development time.

REFERENCES

[1] Henry, P. and Saunders, R. A. *Annals of Clinical Biochemstry,* 1975, **12,** 119.

[2] Richterich, V. R., Greiner, R. and Kuffer, H. *Journal of Clinical Chemistry and Clinical Biochemistry,* 1973, **11,** 66.

[3] Safety of Electrically Operated Laboratory Equipment, Draft U.K. Document (origin SIMA/BSI) for ultimate submission to IEC through SC62D — WG5.

[4] Preparation of Manuals for Installation Operation and Repair of Laboratory Instruments, Sept. 1972, National Committee for Clinical Laboratory Standards, U.S.A.

[5] Roberts, L. B. *Journal of Automatic Chemistry,* 1978, **1,** 32.

